# The role of structural parameters on efficiency and transparency of semi-transparent non-fullerene organic solar cell

**DOI:** 10.1038/s41598-022-19346-7

**Published:** 2022-09-02

**Authors:** Elmira Annabi Milani, Mina Piralaee, Sohrab Ahmadi, Asghar Asgari

**Affiliations:** 1 Faculty of Physics, University of Tabriz, Tabriz, 51665-163 Iran; 2 Photonics Devices Research Group, Research Institute of Applied Physics and Astronomy, University of Tabriz, Tabriz, 51665-163 Iran; 3 School of Electrical, Electronic, and Computer Engineering, University of Western Australia, Crawley, WA 6009 Australia

**Keywords:** Optics and photonics, Physics

## Abstract

Semitransparent organic solar cells have become attractive recently because of their photon harvesting in the near-infrared and ultraviolet range and passing in the visible light region. Semitransparent organic solar cells with ITO/ZnO/PBDB-T:ITIC/MoO3/Ag/MoO3 structure have been studied in this work and the effects of PBDB-T:ITIC active layer thicknesses and the transparent top electrode, MoO_3_/Ag/MoO_3_, thickness on the solar cell performance such as I-V characteristics, the power conversion efficiency, the average visible transmittance, and the color coordinates in the CIE color space are investigated. The drift–diffusion model, including the density of exactions, and their displacement is used to model the devices. The model is examined with experimentally reported devices, where there is a very good agreement between them, then is applied to the new structures. The obtained results show that the average visible transmittance of more than 45% is achievable for these structures with reasonable power conversion efficiency.

## Introduction

Solar energy, besides fusion, has the greatest potential to meet global future needs as one of the main sources of renewable energy. Therefore, harnessing the power of the sun with photovoltaic technologies seems to be the only practical response on a large scale to the energy challenge. On the other hand, organic solar cells (OSCs) have many advantages over conventional inorganic cells such as low cost, light-weight, and intrinsic flexibility ^1^, and can be easily produced in thin rolls, any desired color, and can bend and flex in a specific structure or even inside clothing. Integrating flexible OSCs inside clothing, called carry-on photovoltaics, provides a much larger area for integrated photovoltaics than the ever-shrinking portable devices themselves^2^.

Another important property of OSCs is their flexibility at the wavelength range of absorption spectra because the optical band gap of organic semiconductors can be simply tuned. So, OSCs can adsorb the infrared (IR), and ultraviolet (UV) regions and passes through the visible light. Therefore, those can be used as a window. Generally, the main purpose of windows is to provide natural light with a clear vision, which has been achieved by developing a semitransparent organic solar cells (ST-OSC) technology that selectively harvests near-infrared (NIR) and UV light and passes them in the visible light region. The power-production from UV and NIR photons alone leads to a theoretical single-junction efficiency of 21% in transparent structures. To have higher efficiency, ST-OSC requires a high-quality active layer because they are more sensitive to shunt-leakage^3, 4^. Recently, it has been reported that the ST-OSC with 10 nm Ag achieved a PCE of 12.91% which could be in the range of the best-reported performance of ST-OSC^5^.

The thickness of the active layer is important for SC-OSC performance, besides its transparency. The thickness of organic active layers in OSC-based photovoltaic technology is about one hundred nanometers which are sufficient to capture photons. Also, the thickness of each layer is of importance because the wavelength-specific interference pattern of the optical field will vary depending on the exact device architecture^6^.

Despite the many advantages, OSCs are still not efficient enough to compete with conventional energy sources, and their large-scale production process for industrial commercialization must be facilitated^7^.

Numerous studies have been conducted to improve the power conversion efficiency (PCE), such as the acceptance of solvent additives, applying different active layers, the use of thermal or solvent annealing processes, and the use of triple strategy and reverse structure cells that will be seen in future developments probably^8^.

To fabricate an ST-OSC, the light absorption inside the active layer must be precisely adjusted to transmit sufficient visible light^9, 10^. In addition, both electrodes must be transparent. Although in OSC, the amount of interfacial recombination is affected significantly by the electrode choice, a broader range of materials and deposition techniques can be considered for transparent electrodes because the surface recombination at the active layer interfaces is not a dominant recombination mechanism in these systems, therefore, the limitations due to high series resistance decrease^11^. There are several ways to fabricate transparent electrodes. Carbon nanotubes are suitable candidates because they are both cheaper to produce and have good mechanical flexibility^12^. Another carbon-based solution, graphene, has also been studied and used as the top and bottom electrode in semi-transparent organic solar cells. However, these electrodes have low conductivity and require additional chemical doping^13, 14^. Single or multiple pairs of one-dimensional photonic crystals can also be used as the top electrode to achieve an efficiency of about 5–6% in the average visible transmittance (AVT) of 25%, of which deposition of multiple layers of metal oxide (MoO_3_ or WO_3_) /LiF pairs is necessary^15, 16^. Depending on applications such as sunglasses, building windows, tinted car windows, etc.…, AVT can vary from about 100% to 25% ^17^. Another possible choice for the upper electrode is the dielectric/metal/dielectric (D/M/D) structure. Due to its relatively simple construction and simple fabrication technique, it is more useful in comparison to the alternative approaches such as photonic crystals or graphene-based electrodes^18^. These electrodes have been used in this study.

Also, a significant issue in achieving high efficiency in a device is engineering to select the appropriate material to use as the active layer of OSC. Recently, non-fullerene small molecule acceptors have appeared in OSCs as a viable alternative to conventional fullerene acceptors as electron collectors^19–]^^23^. The reason for the superior performance of non-fullerene OSCs is the enhanced optical and electronic properties of non-fullerene receptors, as a result of easy adjustment of molecular energy levels^24, 25^, superior optical absorption properties^26^, the easy synthesis of these materials and therefore low manufacturing costs. As a result of the mentioned features, non-fullerene acceptors compared to fullerene acceptors^27, 28^ have accelerated the improvement of OSC’s performance over the past few years. For example, the non-fullerene acceptor ITIC has shown particularly promising results. This novel small molecule material has a high lying LUMO level (-3.78 eV), which leads to high open-circuit voltage (V_oc_)^29^. and when coupled with a new polymer (PBDB-T), the efficiency is recorded to be more than 11%^30^.

The carrier’s recombination is a major loss mechanism in OSCs that controls their performance. Considering the recent progress in improving the donor and acceptor materials used in OSCs, a detailed study of the optical properties of the device is very important to further understand dominant recombination mechanisms in OSCs. In the study of such systems, generally, just the contribution of bimolecular recombination and in some cases bulk-trap-assisted recombination is considered, therefore, it is more desirable to include the interfacial recombination mechanisms in these types of structures^23^.

In this work, we have theoretically studied and modeled the role of using PBDB-T:ITIC non-fullerene material in ST-OSCs architecture and considered the effects of the active layer and D/M/D layer thickness on the performance parameters and also AVT of the device.

## Method and material

**Figure 1:**
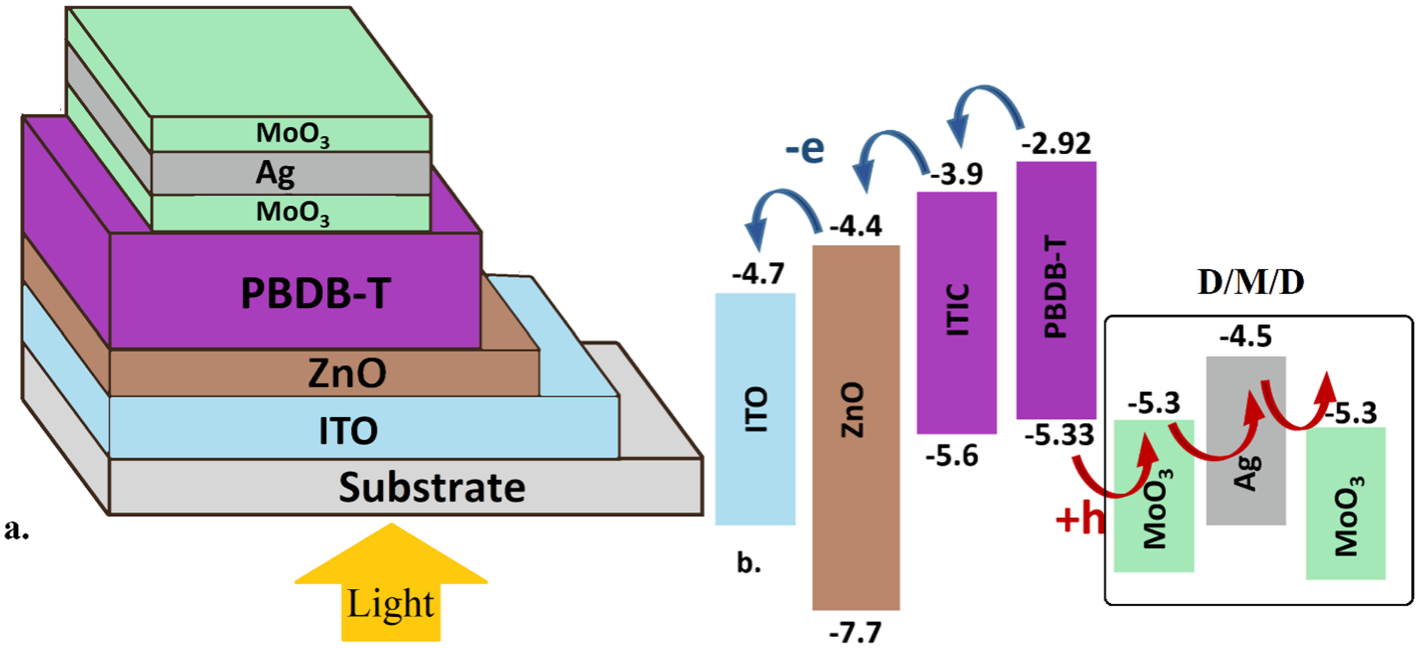
(**a**) Schematic structure, (**b**) The energy level diagram of the materials used in the ST-OSC studied structure ^31^.

The studied structure used for this study, schematically shown in Fig. 1, is the same as the device reported in Ref ^31^ with ITO/ZnO/PBDB-T:ITIC/MoO_3_/Ag/MoO_3_ structure, but with a different active layer and D/M/D layers’ thickness. Where MoO3/Ag/MoO3 acts as the transparent top electrode, the inner MoO_3_ layer also acts as the hole transport layer. Sol–gel processed ZnO layer is used as the electron transport layer and ITO is the transparent bottom electrode.

The combination of PBDB-T and ITIC in the opaque blend structure has previously shown good photovoltaic performance with more than 11% PCE^29^. When it was employed in the semitransparent (ST) structure (Fig. 1a), PCE > 7% was achieved at 25% AVT.

To calculate the device’s performance parameters such as the short-circuit current (J_sc_), the open-circuit voltage (V_oc_), the fill-factor (FF), and the power conversion efficiency (PCE), we used the drift–diffusion model including the density of excitons and their displacement. To calculate the density of electrons, holes, and excitons in the interface regions, we have used the theoretical model based on Koster et al.^32^. The model contains the drift–diffusion of charge carriers and the effect of space charge on the electric field in the device. Recombination is described as a bimolecular process, with the rate given by Langevin^33^.

One of the easiest and most efficient methods for calculating the reflection, transmission, and absorption spectra in optical multilayers is the Transfer-Matrix Method, TMM^34^. we have used TMM to model the whole device transparency, $T(\lambda )$, knowing the transmittance of each layer^6^.

The transparency properties of the device are determined by the transmittance characteristics in the visible light wavelength range (370–740 nm), called the average visible transmittance (AVT), taking into account the photopic response of the human eye $\left(\lambda \right)$ . The AVT value is calculated as follows^35, 36^:

1 $$AVT = \frac{{\int_{370\,nm}^{780\,nm} {S_{AM1.5G}^{{}} \,T(\lambda )\,V_{{}} (\lambda )\,d\lambda \,} }}{{\int_{370\,nm}^{780\,nm} {S_{AM1.5G}^{{}} \,V(\lambda )\,d\lambda \,} }}{,}$$

where ${S}_{AM1.5}\left(\lambda \right)$ is the photon flux under AM 1.5G illumination and $V\left(\lambda \right)$ is the photopic response of the human eyes. AVT value of 25% is an acceptable criterion for window applications where it depends on the devices’ working circumstances^37^. As the window transparency depends on the human eye’s response, the color coordinates (x, y) in the CIE 1931 chromaticity diagram will be another important characteristic of the semi-transparent solar cells. CIE is the most significant system developed by the Commission Internationale de L’Eclairage (CIE, the International Commission on Illumination) to quantify and characterize colors and human perception of color. The color coordinates are calculated using Eq. (2) ^9^:

2 $$x = \frac{{X_{1} }}{{\sum\limits_{i = 1}^{3} {X_{i} } }},\,\,y = \frac{{X_{2} }}{{\sum\limits_{i = 1}^{3} {X_{i} } }}{,}$$

where $X_{i} = \int_{370\,nm}^{780\,nm} {S_{AM1.5G}^{D65} \,T(\lambda )\,\overline{x}_{i} (\lambda )\,d\lambda \,}$, and $S_{AM1.5G}^{D65}$ is the CIE standard D65 illuminant spectrum, and the terms $\overline{x}_{i} (\lambda )\,$ are color-matching functions defined by the CIE protocol (X + Y + Z) = 1.

## Results and discussion

**Figure 2:**
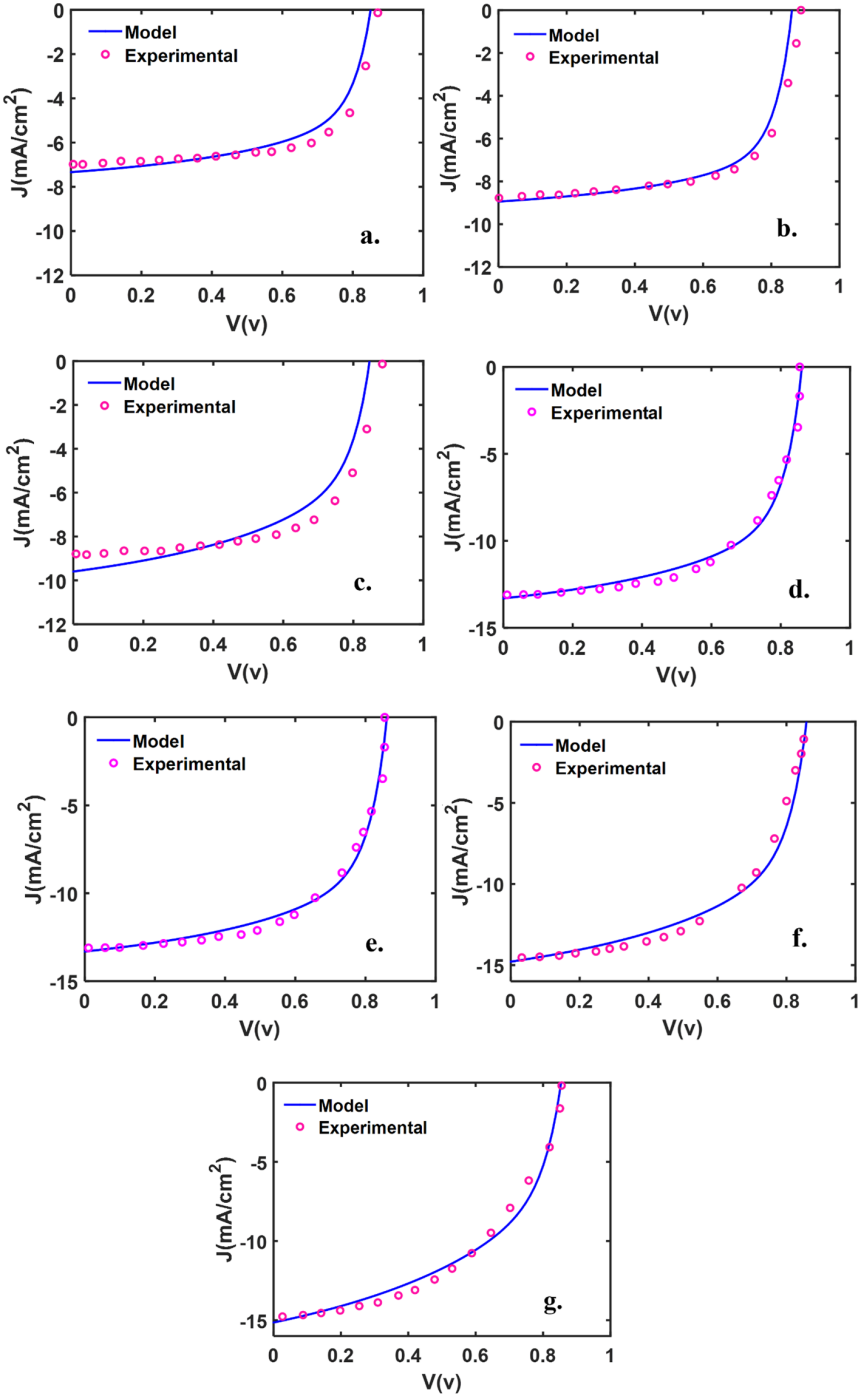
The J-V characteristics of the device with the structure of ITO/ ZnO/ PBDB-T: ITIC/ MoO3/ Ag/ MoO3, with D/M/D thickness of 6 nm/10 nm/40 nm, and PBDB-T: ITIC active layer thickness is: (**a**) 53, (**b**) 59, (**c**) 72, (**d**) 91, (**e**) 100, (**f**) 114, (**g**) 143 nm.

**Figure 3:**
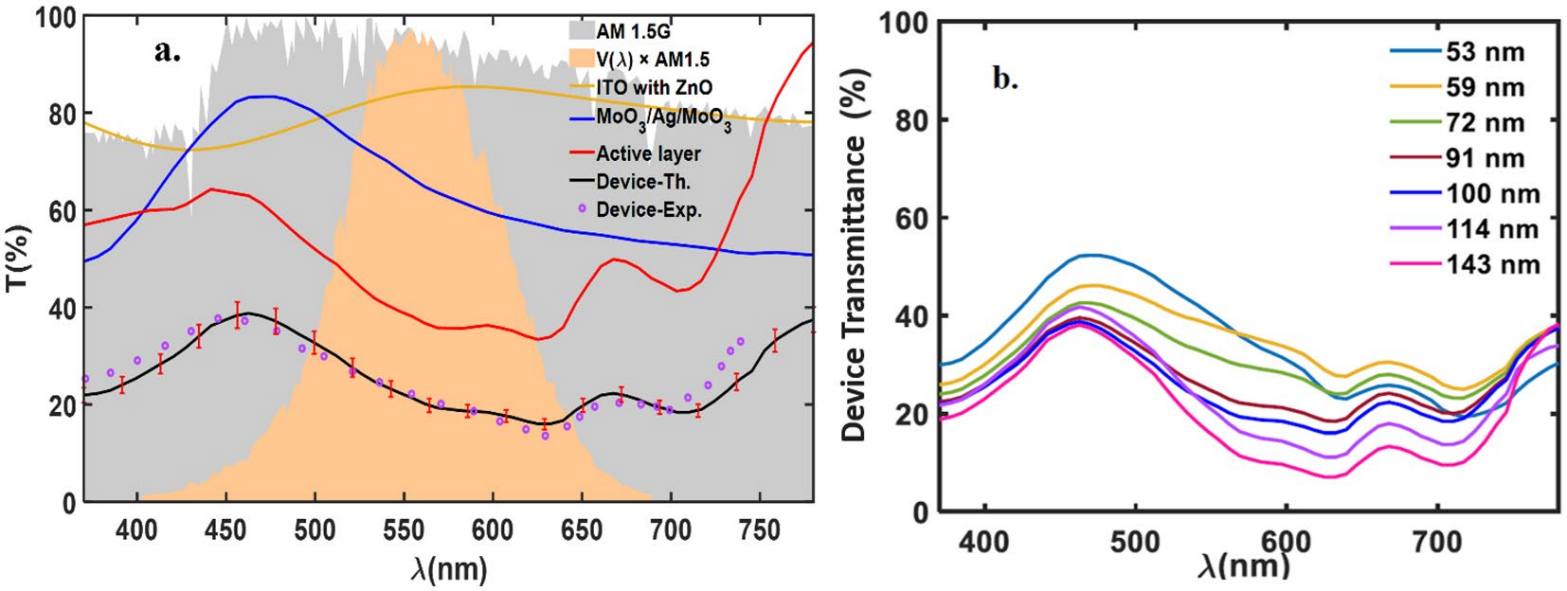
(**a**) The calculated transmittance spectrum of the active layer (100 nm), MoO_3_/Ag/MoO_3_, ITO with ZnO compact layer, the whole device, both theoretical (solid line) and experimental (dotted), AM1.5 spectral irradiance, and $V\left(\lambda \right). {S}_{AM1.5}(\lambda )$. (**b**) The calculated transmittance spectrum of the devices for different active layer thicknesses.

**Figure 4:**
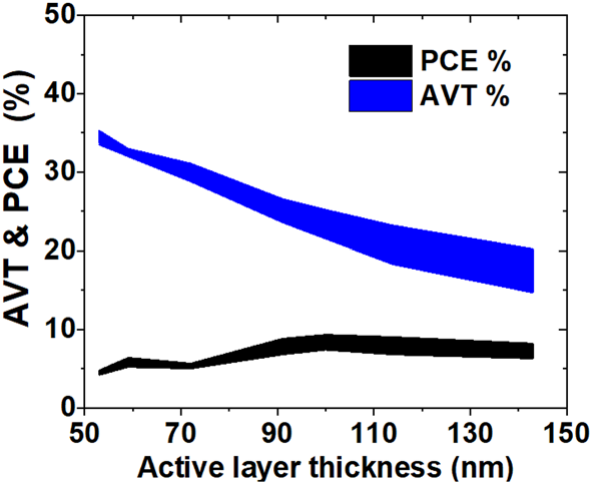
The PCE and AVT, as a function of active layer thickness. The filled area shows the difference between the experimental and theoretical data.

This work has studied ITO/ZnO/PBDB-T:ITIC/MoO_3_/Ag/MoO_3_ structure with different active layer thicknesses and various D/M/D thicknesses. The thicknesses of active layers are 53, 59, 72, 91, 100, 114, 143 nm, and DMD (Top contact) thickness are 10 (nm)/d_m_/30(nm) with d_m_: 4, 6, 8, 10, 12, 14, 16 nm. We first examined our model’s accuracy in comparison with experimental data reported for the device in Ref.^38^, in which the D/M/D thickness is fixed to (6 nm/10 nm/40 nm), and active layer thickness varies from 53 to 143 nm. As shown in Figs. 2, 3, 4, the obtained results are in very good agreement with the experimental results. The characteristics parameters are listed in Table 1. To model the ST-OSC’s performances, one has to know the absorption coefficient of the devices with the structures including different layers, especially for different thicknesses of active layers. For this purpose, we calculated the absorption coefficient as a function of the active layer thickness and fitted it to the absorption coefficient reported experimentally. The fitted relation is presented in Eq. (3), which can predict the absorption coefficient for any thickness of the active layer.

3 $$\alpha_{L} \approx \left( {\frac{{\left( { - 37.12 + 100\lambda } \right) + \left( {0.97 - 3e6\lambda + 2.85e12\lambda^{2} } \right)L}}{{\left( { - 37.12 + 100\lambda } \right) + \left( {0.97 - 3e6\lambda + 2.85e12\lambda^{2} } \right)L_{0} }}} \right) \times \alpha_{{L_{0} }} ;$$

where $\lambda$ is the wavelength, and L is the active layer thicknesses. Knowing the absorption coefficient, $\alpha_{{L_{0} }}$, for an active layer thickness, L_0_, which is reported experimentally, one can find the absorption coefficient for any other thicknesses at different wavelengths.

Figure 2 shows the J-V curve of the devices with structure of ITO/ ZnO/ PBDB-T: ITIC/ MoO3/ Ag/ MoO3, with fixed thickness of D/M/D in 6 nm/10 nm/49 nm, and PBDB-T: ITIC active layer thickness is: a) 53, b) 59, c) 72, d) 91, e) 100, f) 114, g) 143 nm. It is clear from the figures, that there is very good agreement between our model and experimental results. This figure indicates that all devices have the same V_oc_, which is close to 0.85 V. This value is ~ 0.2 V higher than devices containing traditional fullerene acceptors, due to the high LUMO of non-fullerene acceptors^29^. This is a major factor that helps improve this material system's photovoltaic performance. As the active layer thickness was increasing, the J_sc_ also is increasing.

The transmittance spectrum of the ST-OSC for different active layer thicknesses is calculated and compared with those experimental data. As an example, the transmittance spectrum of the ST-OSC with an active layer of 100 nm is presented in Fig. 3a. The figure shows the experimental transmittance for whole devices, besides, the calculated transmittance of the: MoO3/Ag/MoO3 anode, ITO and ZnO compact layer, the active layer, and the whole device. Moreover, for a better understanding of the device’s semi-transparency, AM1.5 spectral irradiance, ${S}_{AM1.5}\left(\lambda \right)$, and ${S}_{AM1.5}\left(\lambda \right)*V\left(\lambda \right)$ are demonstrated. It can be seen that there is a good agreement between the obtained transparency for the device and the experimental data. Also, the error bar is included which shows the model’s accuracy. In the wavelengths of FWHM of ${S}_{AM1.5}\left(\lambda \right)*V\left(\lambda \right)$, the ITO and ZnO compact layer has more than 85% transparency, and the MoO3/Ag/MoO3 anode transparency is about 60% to 75%, and active layer transparency is about 35% ~ 50%.

In Fig. 3b, the calculated transmittance of the ST-OSC for different active layers thickness is presented. As shown in the figure, the transmittance of the ST-OSC with thin active layers thickness (53–72 nm) is higher than 25% at all wavelengths of FWHM of ${S}_{AM1.5}\left(\lambda \right)*V\left(\lambda \right)$, which makes it much suitable for widow application. By exceeding the increment of the active layer thickness, the transmittance of the ST-OSC decreases, whereas, for a longer wavelength, it decreases to less than 25%. However, the AVT of the solar cells in the visible region (370–740 nm) of the devices with active layer thickness thinner than 100 nm is higher than 25% and still suitable for widow application.

In Table. 2, we compared the parameters of the solar cell such as short-circuit current, open-circuit voltage, FF, PCE, AVT, and resistances of the modeled devices with experimental data^31^. In Table.2, **Th.** represents the calculated data, and **Exp.** represents the experimental data reported by ^31^.

As expected and depicted in Table 2 and Fig. 4, with increasing the thickness of the active layer, the photo-absorption increases, and consequently PCE increases. Then, PCE has been decreasing as FF decreases because of the explained recombination effects. FF almost declined with increasing active layer thickness, which is associated with the decrease in shunt resistance values^39, 39^. Although the J_sc_ is highest for the active layer with a thickness of 143 nm, the FF is low and is 49.6%. The optimum PCE is obtained at the active layer thickness of 100 nm with a maximum PCE value of 9.32%.

**Table 1:** Parameters used in the calculation for the device modeling.

Parameter	value
N_A_ (1/cm^3^)	5e25
N_D_ (1/cm^3^)	5e25
N_c_ (1/cm^3^)	8e27
N_v_ (1/cm^3^)	8e27
Electron Lifetime(s)	8.5e-6
Hole Lifetime(s)	7.5e-6
V _built-in_ (V)	1.14
Mobility (cm^2^/V.s)	variable

**Table 2:** The calculated characteristic parameters of the ITO/ZnO/PBDB-T:ITIC/MoO_3_/Ag/MoO_3_ structures with a variation of PBDB-T:ITIC active layer thickness in comparison to experimental data ^31^. MoO_3_/Ag/MoO_3_ thickness is fixed to (6 nm/10/nm/40 nm).

Thickness (nm)	J_sc_ (mA/cm^2^)		V_oc_ (V)		FF (%)		PCE (%)		AVT(%)		R_s_ (Ω.cm^2^)		R_sh_ (Ω.cm^2^)	
	Th	Exp	Th	Exp	Th	Exp	Th	Exp	Th	Exp	Th	Exp	Th	Exp
53	7.33	6.85	0.853	0.884	59.50	66.5	4.79	4.2	33.5	35.3	12.07	13.6	2087	1868
59	8.94	8.41	0.86	0.89	63.95	66.9	6.4	5.2	32.0	33	9.91	15.4	1652	1578
72	9.5	8.75	0.863	0.897	54.75	63.2	5.66	5.01	28.8	31.1	11.82	14	1670	1446
91	13.30	12.62	0.867	0.87	59.57	59.4	8.81	6.8	23.6	26.6	12.92	12.9	1174	1176.3
100	14.28	13.8	0.867	0.886	59	59	9.32	7.4	21.5	25.2	11.73	11.9	990.6	977.4
114	14.76	15.08	0.854	0.87	55.51	51.5	9.02	6.8	18.3	23.2	16.51	15.4	503.2	542.4
143	15.14	13.82	0.854	0.89	49.41	49.2	8.21	6.3	14.7	20.2	19.42	19.5	584.7	582.4

Unlike the PCE, the AVT decreases with increasing active layer thickness. As shown in Fig. 4, and Table 2, for the purposed structures, the devices with PCE of more than 5% and AVT of more than 32% are achievable. The mismatch between experimental and theoretical AVT with increasing the active layer thickness comes from the fitted absorption coefficient (Eq. 3) which has a very low deviation from experimental for thinner active layers.

**Figure 5:**
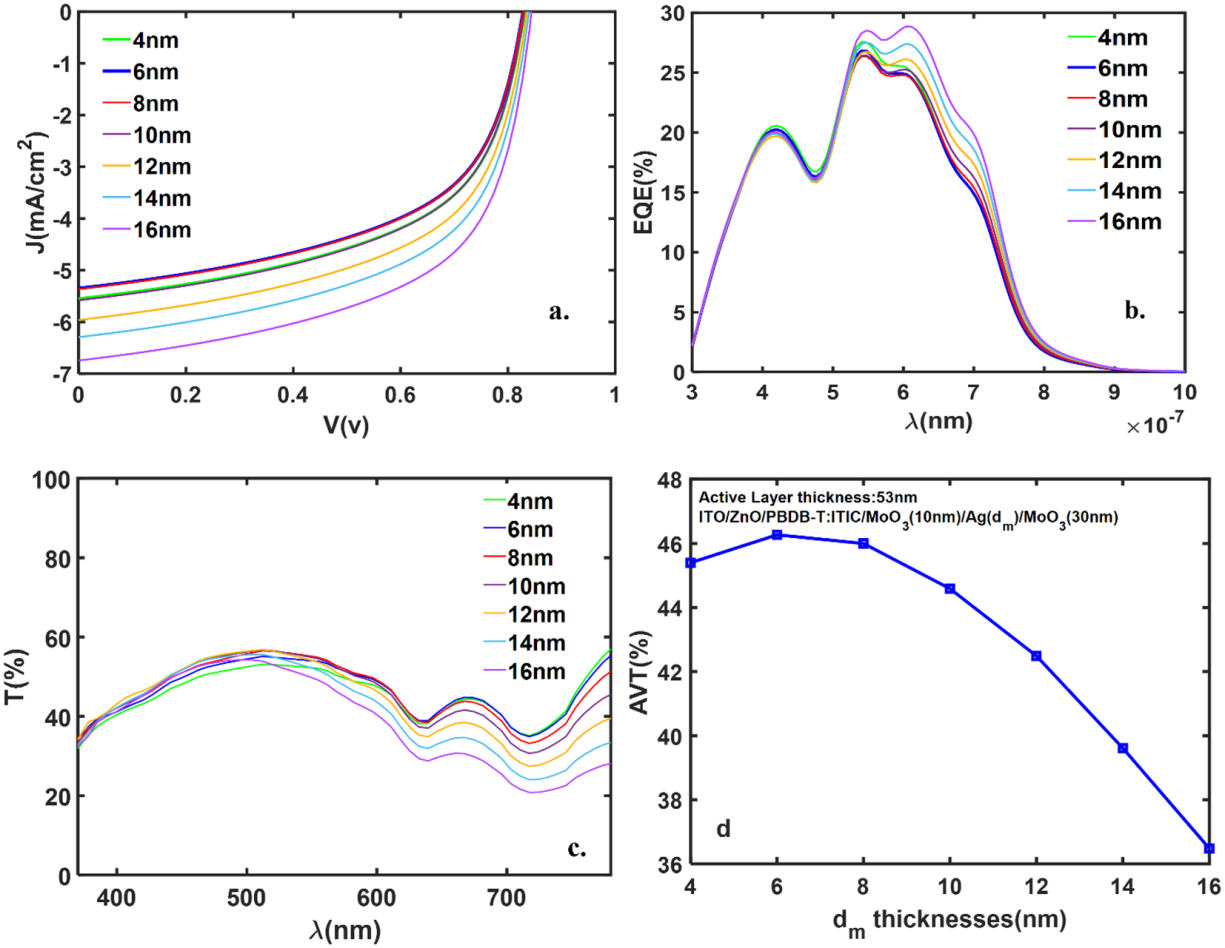
(**a**) The J-V curve, (**b**) the EQE as a function of wavelength, (**c**) T as a function of the wavelength, (**d**) the AVT as a function of d_m_ thicknesses for the devices with the active layer thickness of 53 nm.

It is well known that organic thin-film solar cells act as multilayer optical cavities in which the distribution of the optical field is governed by the effect of optical interference, due to the reflection of the incident light at the layer interfaces ^41^. In the studied devices, the D/M/D top contact which includes 3 layers can be an important multi-layer for optical interference. On the other hand, as the model results for studied structures (Table. 2) are in very good agreement with the experimental data, so the model can be applied to the same structures with different Ag thicknesses in the D/M/D layers. For this purpose, all reported devices in Table 2 have been studied using different metal thicknesses in the D/M/D layer. The considered D/M/D layers has the thickness of MoO_3_(10 nm)/Ag(dm)/MoO_3_(30 nm) with d_m_ = 4, 6, 8, 10, 12, 14, 16 (nm). Using the theoretical model previously explained, all performance parameters such as the J-V curve, EQE, T, AVT, and color coordinates are calculated. As an example, for the devices with the active layer thickness of 53 nm, and d_m_ = 4, 6, 8, 10, 12, 14, 16 (nm), the performance parameters are presented in Fig. 5. As shown in Fig. 5a, with increasing the metal thickness, J_SC_ increases, but the V_OC_ does not change. In these devices, the exciton generation rate depends on the optical field intensity which is located close to the anode/active layer interface when light enters through the D/M/D electrode under top illumination^42^. So, the metal thickness ‘d_m_’ changes can dominantly affect the J_SC_ values. Figure 5b shows the EQE of the devices as a function of wavelengths, in which the highest EQE value belongs to thick metal layers, d_m_ = 16 nm. For all devices, the transmittance is higher than 25% for most visible wavelengths (Fig. 5c). Finally, Fig. 5d shows the AVT of the devices as a function of metal thickness. The AVT of all devices is higher than 36% and the maximum AVT is obtained for d_m_ = 6 nm. So, all devices can be used in the windows application.

All performance parameters for the device are presented in Table 3. As depicted in the table, all devices are practical for the window application with different PCE, while the highest value for PCE is for d_m_ = 16 nm. Also, the highest AVT was achieved for d_m_ = 6 nm, with 3.11% PCE.

**Table 3:** The calculated characteristic parameters of the ITO/ZnO/PBDB-T:ITIC/MoO_3_/Ag/MoO_3_ structures with D/M/D layers thickness of (10 nm/d_m_/30 nm). The thickness of PBDB-T:ITIC active layer is fixed at 53 nm.

d_m_ (nm)	J_SC_ (mA/cm^2^)	FF(%)	V_oc_ (V)	PCE(%)	AVT(%)
4	5.55	55.49	0.82	3.27	45.40
6	5.35	54.66	0.82	3.11	46.27
8	5.36	54.60	0.82	3.10	46.00
10	5.58	55.28	0.82	3.28	44.59
12	6.70	57.86	0.84	4.19	42.49
14	5.96	55.68	0.84	3.59	39.61
16	6.74	57.98	0.84	4.22	36.48

**Figure 6:**
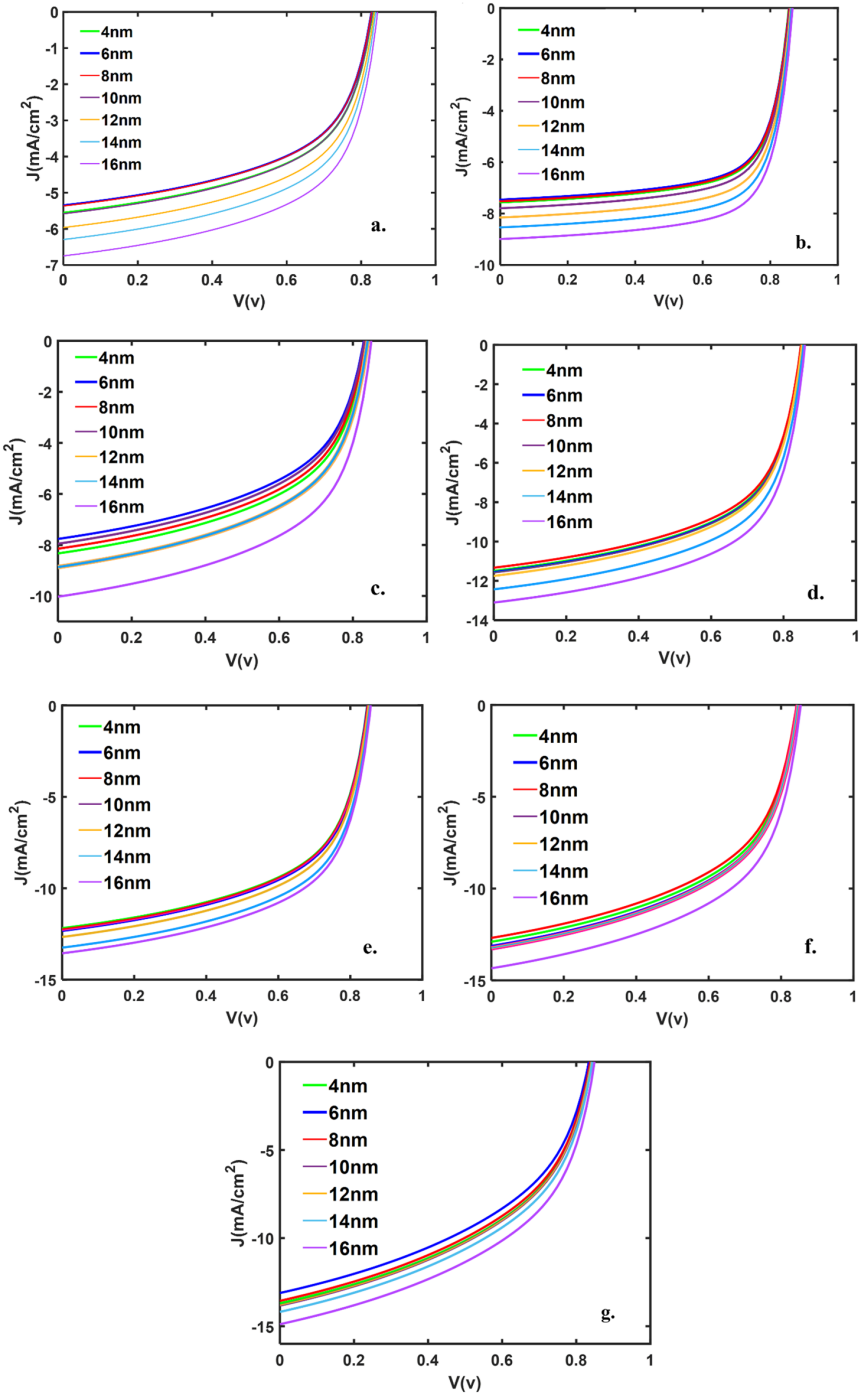
J-V curve for devices with a thickness of active layer (**a**) 53 nm, (**b**) 59 nm, (**c**) 72 nm, (**d**) 91 nm, (**e**) 100 nm, (**f**) 114 nm, (**g**) 143 nm, for various ‘d_m_’ thicknesses.

In Fig. 6, we have presented the J-V curves of the devices with different active layer thicknesses and various d_m_ thicknesses. The figure shows that any change in d_m_ thickness doesn’t change the V_OC_ values. The J_SC_ increases with increasing d_m_ and reaches 14.88 mA/cm^2^ for the sample with an active layer thickness of d_m_ = 16 nm, where the fill factor is the lowest valve (46%-48%) in comparison with other samples. From the point of view of the fill factor, the sample with d_m_ = 16 nm and the active layer of 59 nm has the maximum FF, 70.49%.

**Figure 7:**
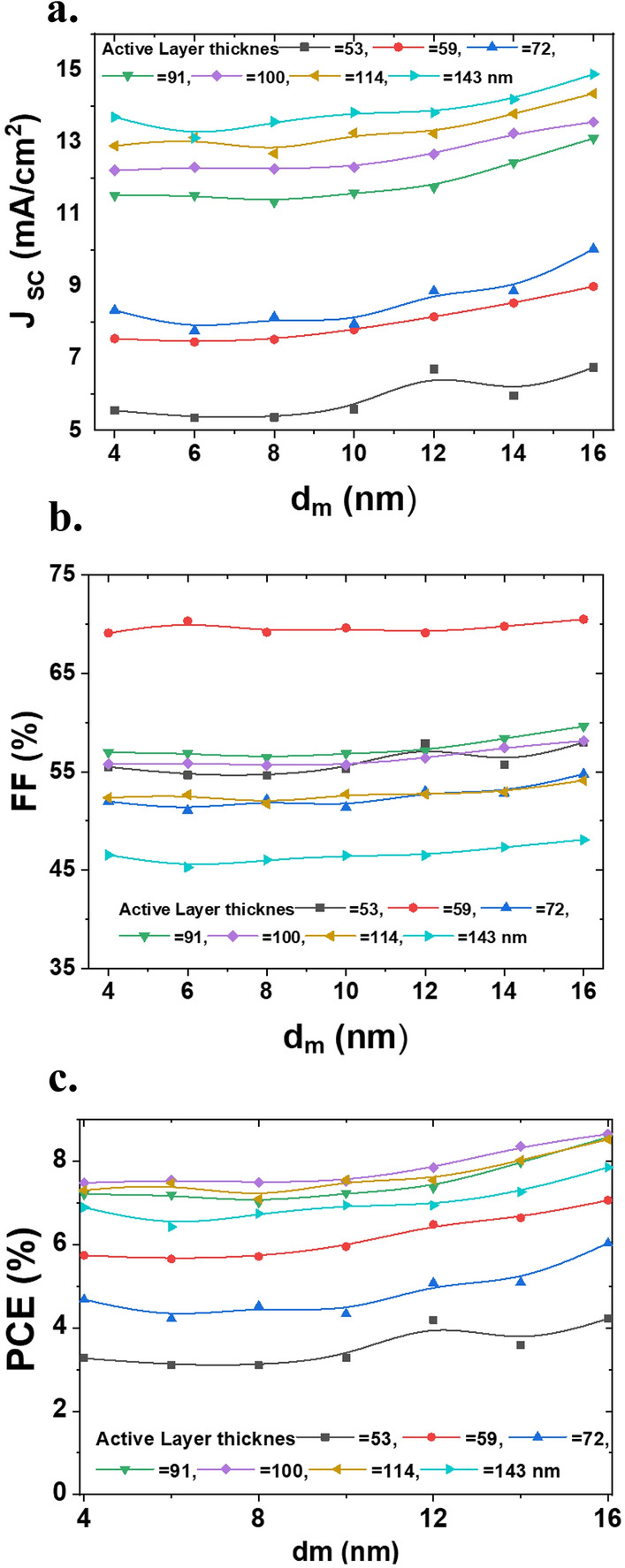
(**a**) The J_SC_, (**b**) FF, and (**c**) PCE (%) of the devices as a function of active and ‘d_m_’ layer thicknesses.

In Fig. 7, the devices’ J_SC_, FF, and PCE are presented as a function of ‘d_m_’ thickness and for various active layer thicknesses. As shown in the figure, for any fixed active layer thickness, with increasing ‘d_m_’, the J_SC_ is increasing slightly, and the FF is almost constant, so, the PCE increases slightly. With increasing the thickness of active layers for any fixed ‘d_m_’, the J_SC_ is increasing, and the FF hasn’t any certain functionality, then, the PCE increases and reaches a maximum, decreases. The maximum value of PCE happens for samples with an active layer of about 100 nm. For devices with a thicker active layer (more than 100 nm), the FF was lower due to the increase in series resistance, which could be due to the distorted distribution of exciton generation within the active layer (most excitons are generated near the anode/active layer interface) and subsequent carrier transport towards respective electrodes.

**Figure 8:**
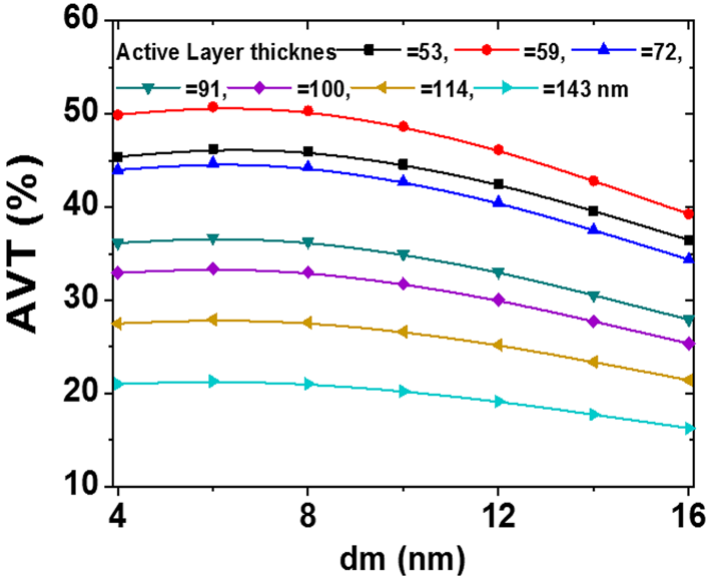
The AVT (%) of the devices as a function of active and ‘d_m_’ layers thicknesses.

To show the applicability of the studied devices in the windows application, the AVT values of the devices are shown in Fig. 8. By increasing the active layer thicknesses, the AVT is increasing, then decreases almost linearly. The maximum value for the AVT belongs to the ST-OSC with the active layer thicknesses of 59 nm (see Fig. 7c). As shown in the figure, for a fixed active layer thickness, the AVT value has a maximum at d_m_ = 6 nm, then with increasing the d_m_ value, the AVT decreases. The figure shows that all devices with different d_m_ and active layer thicknesses thicker than 114 nm deserve semitransparent solar cell conditions.

**Figure 9:**
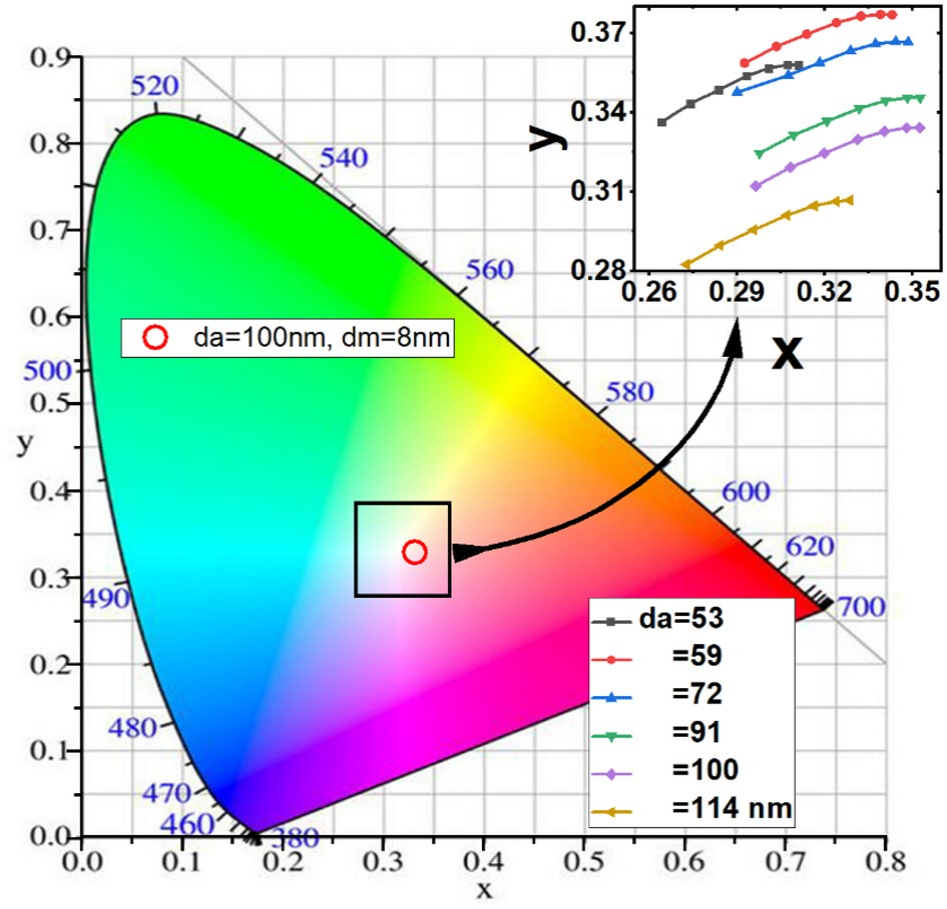
Representation of the color coordinate (x, y) of the ST-OSC with different active layers and d_m_ of D/M/D layer thicknesses, under standard D65 illumination light source on the CIE1931 color space.

The CIE color space, including the coordinates of ST-OSC consisting of different active layer thicknesses and different ‘d_m_’, is shown in Fig. 9. The color coordinates of translucent OSCs with an active layer thickness of about 90- 100 nm are located close to the color point or so-called "white dot" in the CIE chromaticity diagram. Proximity indicates that there is a good achromatic or neutral color sensations when looking through devices under AM1.5G illumination. Hence, these devices can transmit high-quality light with near white sensation to the human eye without changing the original color of an object. However, as the thickness of the active layer changes, the color coordinates move in different directions from the white dot. Also, the coordinates are sensitive to d_m_ values, and both coordinates x and y increase with increasing d_m_ (see the inset of Fig. 9). For a device with the best PCE and AVT, the thickness of the active layer is 100 nm and the color coordinates are slightly away from the achromatic point, however, the device does not alter the transmitted light by a large extent.

## Conclusion

Semitransparent organic solar cells with an active layer of PBDB-T:ITIC and the top transparent electrode of D/M/D are studied. The effects of active layer thicknesses and metal (Ag) layer thickness in the top electrode on the solar cell performance are achieved. The results show that the devices with an active layer thickness of 100 nm have the highest PCE values regardless of any metal layer thickness in the D/M/D layers; the devices with an Ag layer thickness of 6 nm in D/M/D layers have the highest AVT values regardless any active layer thickness. The increasing metal layer thickness in D/M/D layers decreases the AVT and increases slightly the PCE values. For all devices with an active layer thickness thinner than 100 nm with any reported Ag layer thickness, AVT is higher than 25%, and the color coordinates of all these semi-transparent devices are close to the achromatic point. The devices with an active layer thickness of 53 nm and metal layer thickness of 6 nm, have AVT of more than 46%, and PCE of more than 3%.

## Data Availability

The datasets used and/or analysed during the current study available from the corresponding author on reasonable request.
